# CRISPR/Cas9 Application for Gene Copy Fate Survey of Polyploid Vertebrates

**DOI:** 10.3389/fgene.2018.00260

**Published:** 2018-07-20

**Authors:** Fanqian Yin, Wenfu Liu, Jing Chai, Bin Lu, Robert W. Murphy, Jing Luo

**Affiliations:** ^1^School of Life Sciences, Yunnan University, Kunming, China; ^2^State Key Laboratory for Conservation and Utilization of Bio-Resources in Yunnan, Yunnan University, Kunming, China; ^3^State Key Laboratory of Genetic Resources and Evolution, Yunnan Laboratory of Molecular Biology of Domestic Animals, Kunming Institute of Zoology, Chinese Academy of Sciences, Kunming, China; ^4^Centre for Biodiversity and Conservation Biology, Royal Ontario Museum, Toronto, ON, Canada

**Keywords:** polyploidy, CRISPR/Cas9, gene editing, gene-fate, gene copies

## Abstract

Polyploidization occurs widely in eukaryotes, and especially in plants. Polyploid plants and some fishes have been commercialized. Typically, severe genomic perturbations immediately follow polyploidization and little is known about how polyploid offspring survives the genetic and epigenetic changes. Investigations into this require the identification of genes related to polyploidization and the discrimination of dosage-balance from paternal and maternal copies, and regardless of the mechanism being either autopolyploidization or allopolyploidization. New approaches and technologies may discern the mosaic of novel gene functions gained through the recombination of paternal and maternal genes in allopolyploidization. Modifications of Clustered Regularly Interspaced Short Palindromic Repeats (CRISPR) with CRISPR-associated system (Cas) protein 9 (CRISPR/Cas9) have been employed in studies of polyploidization of plants. However, the approach has seldom been applied to polyploidization in vertebrates. Herein, we use CRISPR/Cas9 to trace gene-fate in tetraploid goldfish, and specifically to identify the functional differentiation of two divergent copies of *fgf20a*, which are expressed differently throughout embryonic development. We expect this gene editing system will be applicable to studies of polyploids and the genetic improvement of polyploid livestock.

## Introduction

Polyploidization via whole genome duplication (WGD) involves the integration of more than two complete sets of chromosomes in a cell, either within two different individuals of a species or between two different species. Polyploidization, which occurs most commonly in angiosperms (Otto and Whitton, 2000; Chen, 2007), has generated many crops and fiber corps (International Rice Genome Sequencing Project, 2005; Brenchley et al., 2012; Zhao et al., 2012; Guan et al., 2014; Zhang et al., 2015). In contrast to plants, only a few vertebrate lineages, such as some species of sturgeon, cyprinids, amphibians, and reptiles have polyploid species, as do a few insects (International Rice Genome Sequencing Project, 2005; Brenchley et al., 2012; Zhao et al., 2012; Guan et al., 2014; Zhang et al., 2015).

WGD immediately doubles genetic material and allows for *de novo* functions to develop. However, WGD is usually disadvantageous and rarely advantageous. It increases cell volume and architecture simultaneously, and consequently changes the relationship between the bi- and tri-dimensional components within cells (Comai, 2005). Subsequent difficulties in mitosis and meiosis may cause aneuploidy, which occurred in autotetraploid budding yeast during mitosis (Mayer and Aguilera, 1990). The vast genetic and epigenetic instabilities in both autopolyploidization and allopolyploidization result in offspring facing the polyploidization syndrome called genome shock (Gebler et al., 2017). This results from both cellular and molecular instabilities, including genomic structural changes, chromosomal imbalances, and regulatory incompatibilities, which ultimately result in reproductive failure. These changes can include random gene loss and rearrangement, extremely accelerated mutations (Liu et al., 2016), imbalanced chromosomal rearrangements, and failed paring of homologous chromosomes. Together, these changes result in offspring differing significantly from their progenitors. Assuming that progenitor expression patterns are adaptive, offspring genomics could drive instabilities in cell architecture and genomic regulatory networks, thus leading to deleterious chaos displayed as dosage imbalances and abnormal expressions (Kim et al., 2012; Ng et al., 2012; Zhou et al., 2015). Moreover, allopolyploid offspring must overcome alien genomic fusion (Mayer and Aguilera, 1990). Allopolyploid plants can use heterosis to overcome the synthetic effects of alien genomic fusion (Ni et al., 2009). However, hexaploid individuals (3n = 6x = 150–162 chromosomes) in one population of the *Carassius auratus* species complex occur naturally and they can endure polluted water better than tetraploid individuals, although the reason for this remains unknown. Notwithstanding, WGD may provide evolutionary potential that outweighs genome shock syndrome.

How do polyploid offspring survive genomic shock? Is bigger really better? The initial stage after polyploidization is crucial and dynamic, genetic and epigenetic changes provide some flexibility for survival and reproduction of the neopolyploids. Subsequent fixation of genomic changes may further facilitate survival. However, little is known about which change(s) is(are) crucial for survival and reproduction, and which ones have the potential for further adaption. Further, a knowledge-gap exists regarding how the new genome becomes stable and later on how diploidization reoccurs. Although, new genomic technologies facilitate investigations into polyploidization, explorations into crucial networks or genes await the development of new methodologies into functional consequences. The CRISPR/Cas9 gene editing system can advance fine-scale observations into many issues of polyploidization.

Better than traditional breeding strategies, CRISPR/Cas9 genome editing is a precise, rapid, and cost-effective tool for enhancing production. In plants, CRISPR/Cas9 has generated long-shelf life tomato lines (Yu et al., 2017), produced tomato plants resistant to powdery mildew (Nekrasov et al., 2017), regulated multilocular silique development in cultivated rapeseed (Yang et al., 2018), reduced flag leaf-size (Tang et al., 2018) or increased weight-gain (Xu et al., 2016) in rice, and improved traits in other polyploid crops (Weeks, 2017). In animals, CRISPR/Cas9 has improved muscle-growth performance in pigs (Wang K. et al., 2017) and created favorable traits in other livestock (Ruan et al., 2017). Polyploidization has played a key role in horticulture (e.g., it increases the size of plant organs, buffers deleterious mutations, increases heterozygosity and heterosis; Sattler et al., 2016) and has shown advantages in animal breeding, especially for aquatic organisms (Song et al., 2012). Because polyploid species possess multiple copies of a single genome, the precise editing of each copy from same gene is challenging. However, CRISPR/Cas9 has been used successfully to manipulate gene copies that contribute to economically important traits such as coat-color in domestic sheep (Zhang et al., 2017). So far, CRISPR/Cas9 has been used in studies of the axolotl (Nowoshilow et al., 2018) and *Pleurodeles waltl* (Elewa et al., 2017) to create mutations resulting in the loss of function for the genes *Pax7* and *Pax3*. Notwithstanding, CRISPR/Cas9 remains to be used to explore the functional fates of duplicated gene copies. Herein, we review how to apply CRISPR/Cas9 to efficiently edit the genomes of natural polyploid goldfish to advance breeding and extend the technology to produce synthetic polyploid animals.

## Exploring the Destiny of Gene Copies in *C. auratus*

### Experimental Design

The CRISPR/Cas9 gene editing system uses the Cas9 protein, CRISPR-derived RNA (crRNA) and *trans*-activating crRNA (tracrRNA). Cas9 is formed by the nuclease (NUC) and recognition (REC) lobes. The combination of crRNA and tracrRNA, termed single guide RNA (sgRNA), works well (Nishimasu et al., 2014). Compared to the first and second gene editing techniques, CRISPR/Cas9 is more efficient, more specific, and more convenient in performance, and it has been applied widely in plants and animals. Naturally occurring off-switches in the CRISPR/Cas9 system have been identified, and these may enhance its application in gene editing (Pawluk et al., 2016).

CRISPR/Cas9, as a powerful tool for gene editing, can potentially discern the fate of gene copies (e.g., functional differentiation, change of expression) in polyploids. In polyploid species, gene editing has improved crops (Wang et al., 2014; Lawrenson et al., 2015; Wang W. et al., 2017). Little research has focused on the fate of homologous genes (Endo et al., 2015; Morineau et al., 2017; Nakasuji et al., 2017; Wang W. et al., 2017). One study evaluated the fate of gene copies of polyploidy sub-genomes of cotton (Li et al., 2017), no analyses exist for polyploidy animals. The *C. auratus* species complex includes tetraploid, hexaploid, and octaploid populations. Goldfish (*C. auratus* red var.), a red variant tetraploid with 100 chromosomes, may have experienced allotetraploidization about 10–12 million years ago, and thus each fish has two diverged copies of genes and as many as four alleles (Ma et al., 2014). Our analyses of genomic and transcriptomic data from goldfish obtains divergent patterns of expression for many gene-pairs. Changes such as expression in different tissues or developmental stages, or extremely low levels of expression, indicate the potential sub-functionalization, neo-functionalization, or loss of function. For example, copies *fgf20a*-1 and *fgf20a*-2 expressed in embryonic *C. auratus* have different patterns of expression throughout early development. Copy *fgf20a*-1 has its highest expression at the 14-somite stage (18 hr post-fertilization), then dramatically decreases afterward, yet *fgf20a*-2 slowly increases starting around this stage and finally peaks at the pectoral-fin stage of hatching (after 64 hr). These patterns suggest the occurrence of sub-functionalization.

**FIGURE 1:**
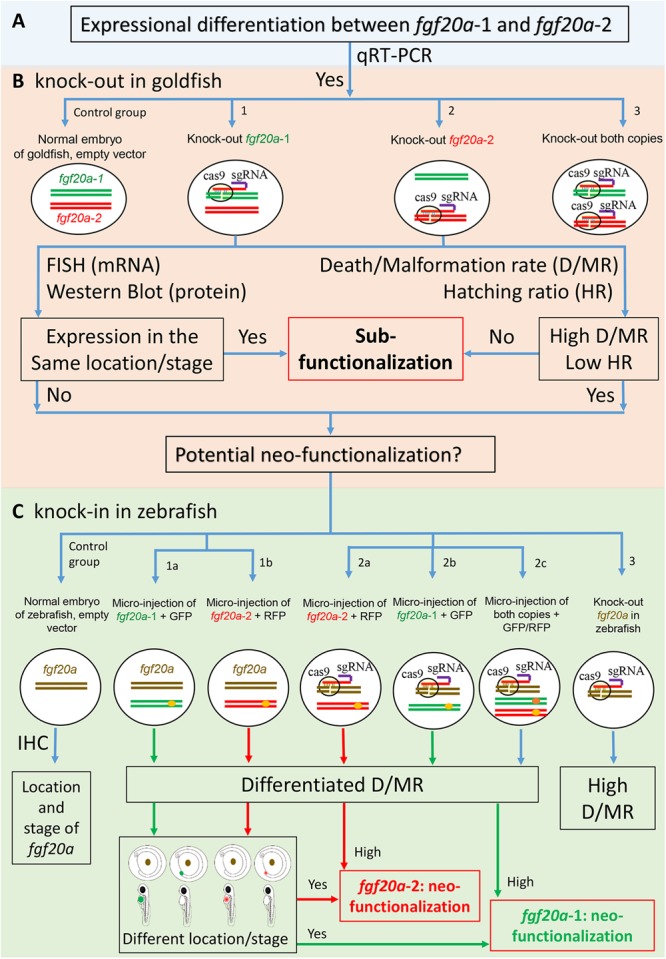
Using CRISPR/Cas9 to confirm gene-fates of two *fgf20a* copies in goldfish and zebrafish embryos. **(A)** qRT-PCR verifies the mRNA expression pattern of *fgf20a*-1 and *fgf20a*-2. **(B)** Four groups of goldfish embryos: the control with normal embryo and empty Cas9 vector, **(B-1)** knock-out of *fgf20a*-1, **(B-2)** knock-out of *fgf20a*-2, **(B-3)** knock-out both copies. Next, *in situ* fluorescent hybridization (FISH) and western blot serve to verify expressional location of mRNA and expression of protein. Death/malformation rate (D/MR) and hatching ratio (HR) of embryos are recorded throughout developmental stages. If knocking-out a single-copy of *fgf20a* (**B-1** or **B-2**) group leads to the other copy expressing in the same location or stage and with no changes or slightly high D/MR and low HR, then sub-functionalization of either *fgf20a* copy exists. If not true, then the result indicates neo-functionalization, which requires further verification by comparing to the single-copy gene in ancestor. **(C)** Verification of neo-functionalization of *fgf20a* copies. Normal embryos of zebrafish serve as the positive control and knock-out *fgf20a* of zebrafish as the negative control. Modified zebrafish embryos with a goldfish gene-copy has two groups: one retains the *fgf20a* of zebrafish and has a micro-injection of **(C-1a)** *fgf20a*-1 + GFP and **(C-1b)** *fgf20a*-2 + RFP; the other has knock-out *fgf20a* with micro-injection of **(C-2b)** *fgf20a*-1 + GFP, **(C-2a)** *fgf20a*-2 + RFP, **(C-2c)** both copies + GFP/RFP. Immunohistochemistry (IHC) and fluorescent fusion protein (*fgf20a*-1 + GFP and *fgf20a*-2 + RFP) serve to verify and locate expression of the proteins. D/MR and HR values can verify whether the knock-in of goldfish markers into zebrafish embryos work or not. If the modified and knock-out *fgf20a* of zebrafish have similar embryonic deficiency rates **(C-3)**, then the knock-in of goldfish marker is not practical. However, if the modified embryos have different rates of death/malformation, then the experiment is feasible. Neo-functionalization of one copy will exist if the expression of either *fgf20a*-1 **(C-1a)** or *fgf20a*-2 **(C-1b)** occurs in a different location or stage (compared to *fgf20a* of zebrafish) and the knock-out *fgf20a* **(C-2a,b)** has high D/MR and low HR values.

CRISPR/Cas9 using knock-in and knock-out genes can verify gene-fates. To do this, an initial qRT-PCR serves to verify the mRNA expression patterns of *fgf20a*-1 and *fgf20a*-2 (**Figure 1A**). After confirmation, it is necessary to establish four groups of embryonic goldfish, including two control groups of normal embryos with and without vectors (**Figure 1B**) and three CRISPR/Cas9-modified groups (**Figure 1B**): (1) knock-out *fgf20a*-1 (**Figure 1B-1**); (2) knock-out *fgf20a*-2 (**Figure 1B-2**); and (3) knock-out both copies (**Figure 1B-3**). *In situ* fluorescent hybridization and western blots can detect the location of mRNA and the expression of proteins, respectively, during developmental stages between segmentation and hatching. Sanger sequencing verifies successful knock-out by detecting frameshift indels around target sites within *fgf20a* copies. Comparisons of death/malformation rates (D/MR) and hatching ratios (HR) would be necessary, because the loss of both copies might be lethal. Assessment initially knocks-out one copy of *fgf20a* (**Figure 1B-1** or **1B-2**). If the alternative copy is expressed in the same location or stage as the knock-out copy, and at the same time shows no changes or a slightly higher D/MR and lower HR compared to the control, then sub-functionalization has occurred. Alternatively, if the remaining copy is expressed in a different location/developmental stage and has a relatively higher D/MR and lower HR than the control, then neo-functionalization is likely.

Compared with sub-functionalization, the verification of neo-functionalization is more complicated. The zebrafish system can serve to verify neo-functionalization by comparing its single-copy genes with candidate duplicated genes in goldfish. New patterns of spatio-temporal expression or new functions when compared to the single-copy genes will indicate neo-functionalization of duplicated genes (Ohno, 1970; He and Zhang, 2005; Duarte et al., 2006). Accordingly, normal and knock-in zebrafish embryos having goldfish gene copies can verify embryonic deficiency and yield visual evidence of gene function (**Figure 1C**). Two groups of modified zebrafish embryos with goldfish gene copies are necessary. One group will have the *fgf20a* of zebrafish plus a micro-injection of (1) *fgf20a*-1 (goldfish) coupled with green fluorescent protein (GFP) (**Figure 1C-1a**) plus (2) *fgf20a*-2 (goldfish) coupled with red fluorescent protein (RFP) (**Figure 1C-1b**). The other group will have knock-out *fgf20a* of zebrafish along with micro-injections of (1) *fgf20a*-1 (goldfish) coupled with GFP (**Figure 1C-2b**), (2) *fgf20a*-2 (goldfish) coupled with RFP (**Figure 1C-2a**), (3) both copies coupled with different fluorescent proteins (**Figure 1C-2c**), and (4) no injection as negative control (**Figure 1C-3**). First, immunohistochemistry (IHC) can confirm the location of *fgf20a* in embryonic zebrafish. Next, fluorescent fusion protein (*fgf20a*-1 + GFP or *fgf20a*-2 + RFP) can verify and locate the expression of either gene copy. Considering the feasibility of the experiment, recorded D/MR and HR values can verify whether the knock-in of goldfish markers into zebrafish embryos work or not. If the modified embryos show rates of deficiency as high as knock-out *fgf20a* of zebrafish (**Figure C-3**), or even more, then the experiment involving knock-in of goldfish is impractical. In contrast, different rates of death/malformation in the modified embryos will indicate a feasible experiment. The further comparison of different experiments can help verify the neo-functionalization. Neo-functionalization of one copy will be seen if the expression of either *fgf20a*-1 (**Figure C-1a**) or *fgf20a*-2 (**Figure C-1b**) occurs in a different location or stage than *fgf20a* of zebrafish, along with the occurrence of knock-out *fgf20a* (**Figure C-2a,b**) having relatively higher D/MR and lower HR values than normal embryos of zebrafish.

### Challenges and Solutions

#### Off-Target

Polyploids own duplicated genomes (Otto and Whitton, 2000). This can cause a high risk for off-target hits by similar gene copies. The optimization of sgRNA and Cas9 can solve the problem, as noted below.

##### Optimized sgRNA

Optimized sgRNA could be used in four ways to improve the efficiency of gene editing in polyploids. First, one way is reducing the similarity of target sites between gene copies (Hsu et al., 2013). Second, CasOT (Xiao et al., 2014) and CHOPCHOP (Montague et al., 2014) can serve to optimize base and mismatch numbers of target sites. Third, whole genome sequencing can detect off-target hits of RNAs (Kim et al., 2015). Finally, the EGFP reporting system can filtrate candidate sgRNAs with high efficiency (Zhang et al., 2014).

##### Optimized Cas9

The CRISPR/Cas9 gene editing system has been applied widely to bacteria (Jiang et al., 2013a), human (Cho et al., 2013; Fu et al., 2013; Shalem et al., 2014), zebrafish (Hwang et al., 2013), plants (Jiang et al., 2013b), and other species. Different organisms use different types of Cas9 protein. In the case of *C. auratus*, the Cas9 protein that has success in zebrafish (Chang et al., 2013) may be a good choice because both fishes belong to Cyprinidae, and the zebrafish is a well-known model animal. To enhance the specificity of genome editing, it is possible to combine a Cas9 nickase mutant with pairs of guide RNAs to introduce targeted double-strand breaks (Ran et al., 2013). This strategy could overcome highly similar gene copies when editing polyploid genes.

#### Fragile *C. auratus* Embryos

Hexaploid embryos of *C. auratus* have high rates of deformity and death (Yi et al., 2006; Benfey, 2010). Thus, it is important to choose embryos with normal appearance and activity. The manual injection of components into zygotes is technically demanding and has inherently low throughput. However, electroporation can deliver efficiency CRISPR/Cas9 components (Qin et al., 2015). This also results in less damage to the embryos.

#### Hard to Detect Off-Target

The genome of polyploid *C. auratus* is highly complex in having many gene copies and genes with insertions and/or deletions. This presents a challenge when differentiating between off-target mutations and self-mutations. Two methods may detect off-target occurrences. Whole genome sequencing (Kim et al., 2015) can compare directly genomes before and after gene editing. Alternatively, integrase-defective lentiviral vectors (IDLV) (Wang et al., 2015) can insert into gene editing sites and then be detected by detect sequencing of off-targets. The later method is more likely to be applied in gene editing of polyploids because it can recognize off-target and self-mutation by inserting a flag into off-target editing sites.

## Conclusion and Perspective

CRISPR/Cas9 is the simplest Type II CRISPR system and it has been used widely in basic gene research. It has potential applications in medicine including with cataracts (Wu et al., 2015), myodystrophy (Long et al., 2014), HIV (Liao et al., 2015), malaria (Gantz et al., 2015), and cancer (Gebler et al., 2017). However, off-target hits limit its specificity in editing genes (Kuscu et al., 2014). For CRISPR/Cas9 gene editing in polyploid vertebrates, the complexity of duplicated gene copies and functional evolution creates an even greater challenge. Our review summarizes several strategies that can enhance its efficiency and specificity when investigating polyploid genomes for potentially differential function(s) of duplicated genes. By employing CRISPR/Cas9 and other techniques, such as qRT-PCR, *in situ* fluorescent hybridization, western blots and the GFP reporting system, it is possible to validate the spatio-temporal expression of divergent gene copies. This helps to validate alterations and even identify novel functions within gene-pairs induced via WGD. By verifying gene-fates in natural allopolyploid goldfish, these strategies could be applied to the synthetic allopolyploidy system. The approach could be extended to breeding polyploid livestock and might help overcome lethality or sterility during synthetic breeding. To this extent, the approach could become one of the important breeding methods in the next few decades. We anticipate that improved methodologies will serve to validate function and these will be applicable to studies of polyploids and the genetic improvement of polyploid livestock.

## Author Contributions

FY wrote parts of experimental design and participated in editing. WL and BL wrote CRISPR/CAS9’s challenges and solutions. JC wrote parts of experimental design and perspective. RM edited the entire manuscript. JL wrote introduction and edited the entire manuscript.

## Conflict of Interest Statement

The authors declare that the research was conducted in the absence of any commercial or financial relationships that could be construed as a potential conflict of interest.
